# Gait Detection in Children with and without Hemiplegia Using Single-Axis Wearable Gyroscopes

**DOI:** 10.1371/journal.pone.0073152

**Published:** 2013-09-04

**Authors:** Nicole Abaid, Paolo Cappa, Eduardo Palermo, Maurizio Petrarca, Maurizio Porfiri

**Affiliations:** 1 Department of Engineering Science and Mechanics, Virginia Polytechnic Institute and State University, Blacksburg, Virginia, United States of America; 2 Department of Mechanical and Aerospace Engineering, “Sapienza” University of Rome, Rome, Italy; 3 Department of Neuroscience and Neurorehabilitation, MARlab Movement Analysis and Robotics Laboratory, “Bambino Gesù” Children’s Hospital, Rome, Italy; 4 Department of Mechanical and Aerospace Engineering, Polytechnic Institute of New York University, Brooklyn, New York, United States of America; Glasgow University, United Kingdom

## Abstract

In this work, we develop a novel gait phase detection algorithm based on a hidden Markov model, which uses data from foot-mounted single-axis gyroscopes as input. We explore whether the proposed gait detection algorithm can generate equivalent results as a reference signal provided by force sensitive resistors (FSRs) for typically developing children (TD) and children with hemiplegia (HC). We find that the algorithm faithfully reproduces reference results in terms of high values of sensitivity and specificity with respect to FSR signals. In addition, the algorithm distinguishes between TD and HC and is able to assess the level of gait ability in patients. Finally, we show that the algorithm can be adapted to enable real-time processing with high accuracy. Due to the small, inexpensive nature of gyroscopes utilized in this study and the ease of implementation of the developed algorithm, this work finds application in the on-going development of active orthoses designed for therapy and locomotion in children with gait pathologies.

## Introduction

Disorders of gait affect an estimated 1.1 million children in the United States as of 2007 [1] and may originate from different somatosensory conditions [2]. Many assessment tools, therapies, and rehabilitation strategies for these disorders are impacted by the complex problem of recognizing the repeated features or “phases” of the gait cycle [3]. In fact, detecting gait phases plays an integral role in the design of controllers for many real-time therapies which seek to coordinate body support and limb progression required for locomotion. Therapies which rely on such information include functional electrical stimulation (FES) [4], [5], active orthoses [6]–[9], and behavioural strategies [10]. While current FES therapies actively assist walking, they rely on patients’ manual stimulation for even simple locomotory tasks [11]. The use of gait identification in active orthoses is expected to permit such therapies to operate synchronously and synergistically with patients [8]. Finally, behavioural training based on adherence enhancing strategies needs accurate feedback to obtain gait symmetry [10].

Sensors used in gait detection include accelerometers, gyroscopes, force sensitive resistors (FSRs), capacitive sensors, and composite inertial measurement units (IMUs) [12]–[23]. Computational methodologies for gait phase detection fall into two major categories: algorithms which partition the gait phases through thresholds for sensor data based on experimental observation [12], [17], [20] and machine-learning schemes which extract patterns from large sensor datasets based on a few assumptions [21], [24], [25]. In the first category, data from a variety of sensors may be fused to both partition individual gait cycles and identify phases within one cycle. The second category takes advantage of recent techniques from computer science, which offer methods for pattern recognition in periodic or quasiperiodic gait data, such as: (i) logic networks trained with and used to analyse data from an electroneurogram of the sural nerve [24]; (ii) probabilistic Bayesian networks implemented on video data [25]; and, finally, (iii) hidden Markov models (HMMs) used to identify the most probable gait phase sequence from shoes instrumented with pressure sensors [21] or from single-axis gyroscope data [23].

In a larger research goal, we are developing an active lower limb orthosis for rehabilitation in children with gait pathologies. We expect that the identification of children’s gaits phases could enhance the control of wearable devices, allowing a more efficient functional training and personalization with respect to specific motor abilities. Towards this end, it is essential to identify gait phases in a robust manner from minimal sensor data that can be acquired and processed onboard a mobile device sized for a child.

From this perspective, here, we propose a novel post-processing algorithm for gait detection using single-axis gyroscope data based on a trained HMM [26] whose execution is automated. A HMM-based algorithm is selected over other approaches as it has been previously demonstrated to be successful at identifying motor tasks using wearable and remote sensors [23], [27], [28]. The HMM parameters are acquired directly from experimental data through a single preliminary trial integrating gyroscopes and FSRs for labelling training data; we validate experimental results against the FSR signal. Gyroscopic sensors are selected over FSRs for experimental trials due to their ease of implementation in small-sized, paediatric orthoses. Although FSRs are delicate and require precise positioning on the foot sole, they are only used in a single preliminary trial to calibrate and train the algorithm, after which the real time control of an active orthosis may be achieved with only gyroscopes. We compare results between children with hemiplegia (HC) and a control group of typically developing children (TD).

This strategy is entirely novel in the subjects it targets, since, to the best of our knowledge, current studies on neurologically-based gait pathologies using wearable sensors target only adult participants [22], [29], [30]. Our analytical approach follows the recently published work of Mannini and Sabatini [23], wherein the authors develop a HMM for gait detection from foot-mounted gyroscopes similarly to this study. However, our methodology for algorithm training differs from this previous work, since gait phases in the training dataset are manually identified and the analysis is only used in post-processing in [23]. In contrast, our work synthesizes an automated method for labelling training datasets without operator intervention and can be implemented in real time.

The goals of this study are fourfold. First, we seek to answer the question of whether a gait detection algorithm using an automated HMM on one-dimensional angular velocity data, from sensors placed on the subjects’ feet, is able to generate equivalent results as a reference signal provided by FSRs over a variety of walking and non-walking tasks. Once the HMM algorithm is validated, we explore its ability to differentiate between the gait of TD and HC. Next, we investigate the use of gait detection as an assessment tool for hemiplegia of varying severity by comparing results obtained with assessments generally used in clinical practice. Finally, we introduce and validate a reduced version of the HMM which can be used for real-time processing.

## Materials and Methods

### Ethics Statement

The protocol was approved by the ethics and medical board of the Children’s Hospital “Bambino Gesù”, Rome, Italy. The goals and procedure were explained to the participants and their parents before the experiment started; oral informed consent was obtained from children and written consent was obtained from their parents. Raw data from anonymous subjects is stored at the authors’ institutions and will be provided on request after the approval provided by the ethics board.

### Participants

A total of twenty participants comprising ten TD (9.5±2.0 years, mean ± standard deviation) and ten HC (8.8±3.8 years) were enlisted in the study. TD had no known pathologies influencing their innate walking patterns. HC were able to walk short distances at low speeds either with or without passive assistive devices, such as orthoses, walkers, or crutches. The pathology severity was rated according to the Gross Motor Function Measure (GMFM) [31], see Table 1. The GMFM ranges from 0 to 100, where higher scores indicate more physical ability and, since this rating system has a high inter-rater reliability [31], a single licensed physician scored all the patients in this study.

**Table 1:** Demographic data and experimental details for children with hemiplegia.

Patient number	Age (years)	Affected side	Gross Motor Function Measure^a^	Walking aid used in study
1	5	Both	97.8	None
2	8	Left	57.3^b^	None
3	7	Left	78.6	Foot orthoses
4	12	Right	97.9	None
5	5	Left	86.5	None
6	13	Right	93.3	Foot orthoses
7	10	Left	94.3	None
8	7	Right	70	None
9	5	Both	88.2	None
10	16	Right	86.8	None

a Gross Motor Function Measure GMFM-88 and Classification System.

b Gross Motor Function Measure GMFM-66.

### Hardware

Participants’ lower limb motion was captured using FSRs and IMUs. The FSRs (Wave, Cometa, Italy) measured the contact between each foot and shoe and they communicated wirelessly to a hub. The IMUs (XBus Master MTx, Xsens Technologies, The Netherlands) comprised tri-axial gyroscopes, tri-axial accelerometers, and tri-axial magnetometers. These units were wired to a base station attached to a belt worn by the participant; the base communicated wirelessly using Bluetooth.

In the present study, one-dimensional data from gyroscopes embedded in IMUs were used to measure the angular velocity of the participants’ feet. Each IMU’s y-axis was aligned with the subject’s sagittal plane; alignment precision was verified by manually moving each subject’s feet in the sagittal plane and verifying that the gyroscope output responded to the movement only along the desired axis. The signals from the two types of sensors were synchronized using a data acquisition board (NI USB-6211, National Instruments, USA). Unfiltered data were acquired at 200 Hz from the FSRs and 50 Hz from the IMUs; the IMU data were further interpolated to match the FSR acquisition rate.

### Experimental Protocol

**Figure 1:**
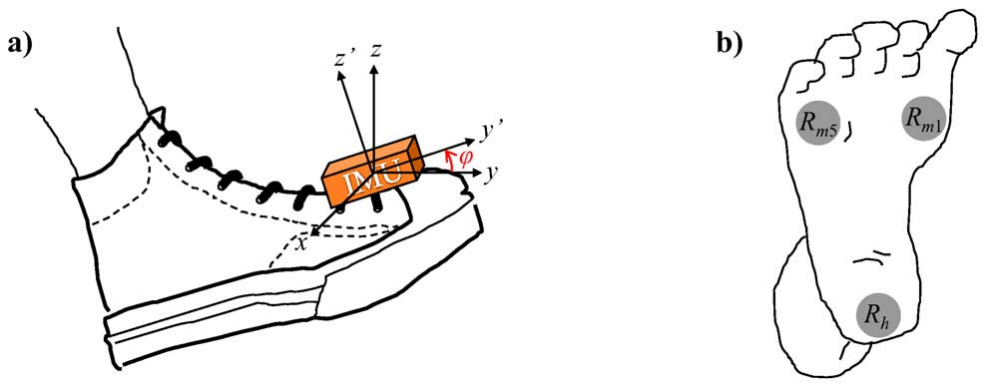
Schematic of positions for IMU and FSRs. a) An IMU is attached to the shoe and φ measures the angular position in the sagittal plane with respect to resting position and b) FSRs are placed on the sole of the foot at the heel and the first and fifth metatarsals.

The participants were equipped with an IMU on each shoe and three FSRs on the foot sole at the heel and at the first and fifth metatarsophalangeal articulations, denoted *R_h_*, *R_m_*_1_, and *R_m_*_5_, respectively, see Figure 1.

After verifying proper threshold values for the FSR-defined reference data and the dominance of gyroscope signals in the sagittal plane, participants were asked to perform four walking tasks and four non-walking tasks. The four walking tasks comprised walking on a treadmill for at least 60 seconds with all combinations of two speeds and two inclinations.

Specifically, we selected: level walking at 1.0 km/h (*L*_1.0_); inclined walking (8.5% inclination) at 1.0 km/h (*I*_1.0_); level walking at 1.5 km/h (*L*_1.5_); and, finally, inclined walking (8.5% inclination) at 1.5 km/h (*I*_1.5_). The treadmill speeds and inclinations were chosen to be performable by all participants, as assessed by a licensed physician.

The non-walking tasks comprised transitioning from sitting with feet on the ground to standing without moving one’s feet (*S*); touching one’s toes from a standing position allowing knee and hip flexion without moving one’s feet (*T*); and completing one revolution clockwise (*CW*) and counter clockwise (*CCW*) without picking one’s feet off the floor. The participants were instructed to keep their feet fixed on the floor during *S* and *T* and to slide them along the floor to turn their bodies during *CW* and *CCW*.

The full set of tasks was completed by all participants except one patient (patient 3), who was unable to perform *T* due to balance limitations. All tasks were repeated two times; the first *L*_1.0_ trial was used as a training trial for acquiring HMM parameters and the second trials of all tasks were analysed as experimental trials. The order of experiments was *L*_1.0,_ *L*_1.5,_ *I*_1.0,_ *I*_1.5,_ *S*, *T*, *CW*, and *CWW* for all HC, with the two trials of each condition completed consecutively, except for patient 3 who, as mentioned before, did not perform the condition *T*.

All trials, including instrumentation, walking and non-walking tasks, and de-instrumentation, were completed within one hour by all participants. HC rested sitting between tasks if fatigue became evident; all TD completed the tasks without expressing fatigue.

### Data Treatment

Data from experimental trials were analysed off-line using MATLAB software (MATLAB 2012a, MathWorks, USA) to generate discrete time series, with a time step of 5 ms, whose elements belonged to the four states of the gait cycle: stance (ST), heel off (HO), swing (SW), and heel strike (HS). These states outline the general progression of a step for a single foot. ST corresponds to the state where the entire foot sole in contact with the floor; HO to the state where the toe, but not the heel, is in contact with the floor; SW to the state where no part of the foot is in contact with the floor; and HS to the state where the heel, but not the toe, is in contact with the floor. We developed a trained HMM algorithm for off-line gait detection in post-processing (A_OL_) and its real-time incarnation (A_RT_).

The reference signal was taken as the voltage from the FSRs, similarly to the experimental methods in [15], [20]. By properly defining thresholds for the signal, the state of each FSR was able to be determined. The four gait phases were defined as follows: ST when *R_h_* was pressed and *R_m_*_1_ or *R_m_*_5_ were pressed; HO when *R_h_* was not pressed and *R_m1_* or *R_m_*_5_ was pressed; SW when no FSRs were pressed; and, finally, HS when *R_h_* was pressed and neither *R_m_*_1_ nor *R_m_*_5_ was pressed.

Algorithm A_OL_ was constructed with a trained HMM based solely on gyroscope data, which was treated before analysis with a low-pass Butterworth filter with 15 Hz cut-off frequency [23]. Similarly to the analysis in [23], the four gait phases were defined as states and the Viterbi algorithm was used to identify the most probable state sequence from the model for an experimental dataset [26].

First, the model was trained on the designated dataset from training trial of *L*_1.0_. The percentage of time spent in each of the four gait phases during each gait cycle was used to create a probability distribution of the states used in the HMM. The gyroscope signal from the training trial was partitioned into individual cycles using the FSR data, that is, without operator intervention. The probability distribution for the initial state was taken as uniform and the state transition matrix of the model was trained on the entire gyroscope time series from the training dataset. The gyroscope data from experimental trials were then analysed with the trained state transition matrix and identified parameters, and the Viterbi algorithm was used to identify the state sequence for this data.

We then adapted this algorithm to enable real-time processing, referred to as A_RT_. In particular, we performed the analysis on the experimental dataset with the same training as in A_OL_, but we used a forward algorithm to identify gait phases as opposed to the Viterbi algorithm. Since this processing does not use a backward step to refine the analysis, it is computationally less heavy than the Viterbi algorithm and it may be performed sample by sample on the experimental signal. Thus, A_RT_ may be implemented in applications requiring real-time gait detection, such as orthoses with active, state-dependent control.

### Statistical Analyses

From both implementations of the algorithm, we generated state time series for the experimental trial of each of the eight tasks. We computed the specificity and sensitivity of gait phases detected by A_OL_ with respect to the reference FSR signal [23], [32]. Following the analysis in [23], these quantities were calculated using a window of 60 ms centred at each time step to define concurrence of transitions between gait phases in the compared signals. For typical gait patterns, at most one gait phase occurs in a time window of this length, which accounts for 6% of a one-second-long step. In the case that no strides were detected by FSRs and A_OL_, which was noted especially during the trials of non-walking tasks, the sensitivity was set to 1 to avoid division by zero.

The statistical significance of variation in these quantities for the different participant populations was computed with a one-way analysis of variance (ANOVA) [33], with specificity and sensitivity as dependent factors and health status, i.e. TD and HC, as independent factor. To compare performance between TD and HC, we performed an ANOVA on the percentage of time spent in each state with health status as the independent factor. Spearman’s correlation coefficient with an *R*-to-t conversion and t-test was used to ascertain correlations between GMFM and experimental performance [33]. All statistical tests were performed with MATLAB and Microsoft Excel (Microsoft Excel 2010, Microsoft, USA) and significance was taken as *p*<0.05. In particular, we used the built-in MATLAB function *corrcoef.m* for the t-test, which computes the following t-statistic to find the statistical significance of a correlation coefficient: t = *R*((*N*-2)/(1-*R* ^2^))^1/2^, with *N* equal to the number of data points. Statistics are reported below as (test, value of F-test statistic, significance).

## Results

### Gait Phases Detected by A_OL_

**Figure 2:**
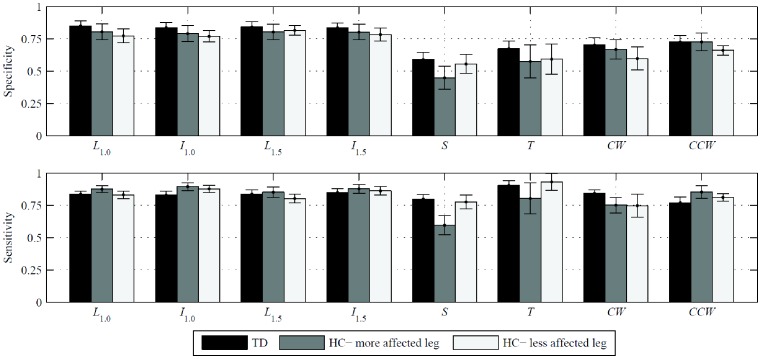
Specificity and sensitivity of A_OL_-detected gait phases, with FSR reference, for typically developing children and children with hemiplegia. The three populations are the combined legs of typically developing children (TD), the more affected legs of the children with hemiplegia (HC), and the less affected legs of the children with hemiplegia. Performance measures are given as mean ± one standard error over each population. For patients with equally affected sides, the right is taken as the more affected leg.

In Figure 2, we report the specificity and sensitivity of the time series computed by A_OL_ with respect to the FSR signal. For all participants in walking tasks, we find consistently high values for both specificity and sensitivity, indicating correspondence between the output of A_OL_ and the reference signal. In particular, the mean specificity and sensitivity are greater than 0.77 for walking tasks averaged over each health status. Moreover, the algorithm performs equally well for TD and the more and less affected legs of HC (ANOVA, F [37], [2]<2.2<3.3 = F_crit_, NS for specificity and sensitivity in all tasks).

### Gait Phase Percentages Detected by A_OL_

**Figure 3:**
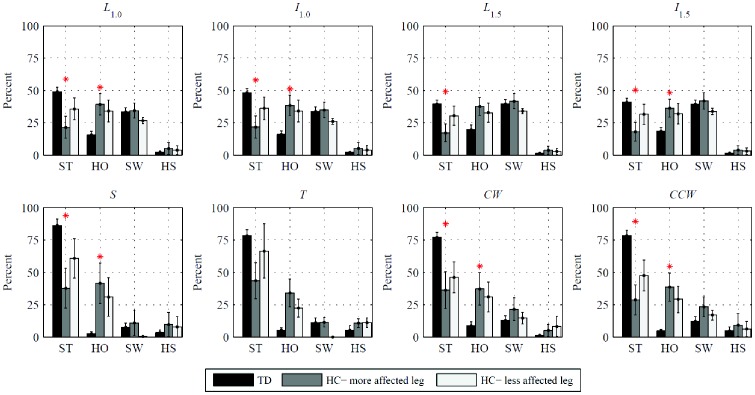
Percent time spent in each gait phase by typically developing children and children with hemiplegia. The three populations are the combined legs of typically developing children (TD), the more affected legs of the children with hemiplegia (HC), and the less affected legs of the children with hemiplegia. Results are given as mean ± one standard error over each population, detected by A_OL_. For patients with equally affected sides, the right is taken as the more affected leg. Gait phases in which the three populations spend statistically significant different times are starred (ANOVA, *p*<0.05).

The percentages of each task spent in the four gait phases grouped by participants’ health status are reported in Figure 3. Gait phases for which the time spent depends significantly on health status of the participant are starred, with results from the ANOVA reported in Table 2. We find significant differences depending on health status in ST and HO for seven and six out of eight tasks, respectively, which reflects toe walking exhibited by many patients with hemiplegia.

**Table 2:** *p* values from ANOVA on time spent in gait phases depending on health status.

Task	ST	HO	SW	HS
***L*** **_1.0_**	4.7 (<0.01)	5.3 (<0.01)	0.8 (0.46)	0.3 (0.73)
***I*** **_1.0_**	4.1 (<0.05)	5.0 (<0.05)	1.0 (0.39)	0.3 (0.72)
***L*** **_1.5_**	3.9 (<0.05)	3.0 (0.06)	0.7 (0.58)	0.5 (0.62)
***I*** **_1.5_**	4.1 (<0.05)	3.3 (<0.05)	0.7 (0.51)	0.4 (0.64)
***S***	5.1 (<0.05)	5.1 (<0.05)	0.6 (0.55)	0.3 (0.72)
***T*** **^a^**	2.2 (0.12)	2.7 (0.08)	0.8 (0.46)	0.2 (0.82)
***CW***	5.3 (<0.01)	3.6 (<0.05)	0.5 (0.60)	0.8 (0.46)
***CCW***	8.2 (<0.01)	7.8 (<0.01)	1.0 (0.37)	0.2 (0.15)

Percent time in each gait phase is the dependent variable and health status is the independent variable, reported as the value of F [37], [2] with the *p* value in parentheses. ^a^F [35], [2], due to patient 3′s lack of completion of task *T.*

### Use of A_OL_ as an Assessment Tool for Gait Pathologies of Varying Severity

Correlations between patients’ GMFM and the time spent in each gait phase are reported in Table 3. For the population of 10 patients, the critical correlation coefficient for statistical significance is *R* = 0.58. For all gait phases in the less affected leg, we see small-magnitude correlations. However, the time spent by the more affected leg in ST and HO shows significant positive and negative correlations with GMFM, respectively, in the majority of walking and non-walking trials. All conditions that are not strictly significant are within 0.04 of the absolute critical value of *R* (0.57 for *L*_1.5_ and *S* in ST, 0.56 for *T* in ST, 0.55 for *CCW* in ST, −0.56 for *L*_1.5_ in HO, −0.54 for *I*_1.5_ in HO).

**Table 3:** Correlation coefficients between GMFM and time spent in each gait phase by children with hemiplegia as determined by A_OL_.

	More affected leg				Less affected leg			
Task	ST	HO	SW	HS	ST	HO	SW	HS
***L*** **_1.0_**	0.60	−0.68	0.06	0.04	−0.06	0.03	0.06	0.04
***I*** **_1.0_**	0.60	−0.66	−0.02	0.04	−0.05	0.04	0.01	0.04
***L*** **_1.5_**	0.57	−0.56	−0.01	0.04	−0.06	0.11	−0.23	0.04
***I*** **_1.5_**	0.59	−0.54	−0.09	0.02	−0.04	0.18	−0.49	0.04
***S***	0.57	−0.76	0.25	0.04	−0.18	0.17	−0.16	0.04
***T***	0.56	−0.76	0.24	0.02	−0.28	0.30	−0.26	0.02
***CW***	0.59	−0.75	0.09	0.04	−0.17	0.25	−0.24	0.04
***CCW***	0.55	−0.77	0.23	0.04	−0.05	0.05	−0.05	0.03

### Matching Results from A_OL_ and A_RT_

**Figure 4:**
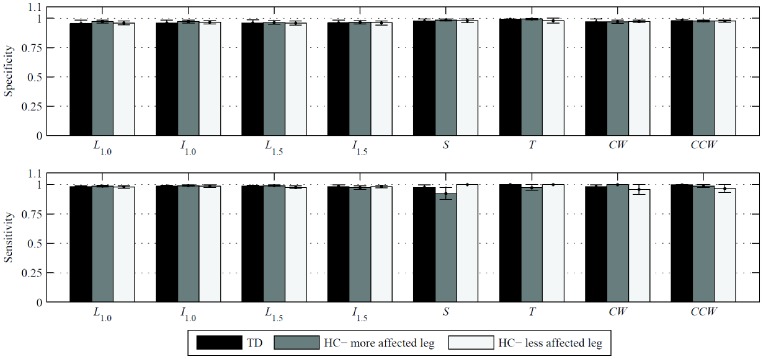
Specificity and sensitivity of A_RT_-detected gait phases, with A_OL_ reference, for typically developing children and children with hemiplegia. The three populations are the combined legs of typically developing children (TD), the more affected legs of the children with hemiplegia (HC), and the less affected legs of the children with hemiplegia. Performance measures are given as mean ± one standard error over each population. For patients with equally affected sides, the right is taken as the more affected leg.

In Figure 4, we report the specificity and sensitivity of the time series computed with A_RT_ using A_OL_ as a reference. We find the minimum mean values for specificity and sensitivity to be very large for all conditions. In particular, the mean specificity and sensitivity are greater than or equal to 0.95 for walking tasks averaged over each health status and are greater than 0.92 for non-walking tasks averaged over each health status.

## Discussion

### Gait Phases Detected by the HMM Algorithm Correspond to those from the Reference Signal

In this study, we find that data from a single-axis gyroscope mounted on the foot is sufficient to detect gait phases in TD and HC, with a prior training phase carried out for each patient using three FSRs added to the sensor set on each foot. From the reference FSR signal, we are able to limit the sensor set in experimental trials to gyroscopes; the HMM consistently detects the gait phase from this data after an initial training. We favour gait detection with a single gyroscope since this sensor is universally easy to use. Although the sensors used here are embedded in IMUs for off-the-shelf convenience, gyroscopes are easily miniaturized and inexpensive and have a high signal-to-noise ratio. Thus, the proposed algorithm has the potential to impact the processing of sensor data implemented in therapies which patients may use independently of clinical supervision.

The developed HMM-based gait detection algorithm offers a viable data analysis protocol for such gyroscope data. Although correspondence between the results of this data treatment and the reference algorithm is less striking than the results from [23], which reports very large values for specificity and sensitivity, the algorithm proposed here requires no direct operator oversight and is easily trained for each individual. Conversely, in the algorithm in [23], gait phases are manually assigned during training, which is time-intensive and user-dependent. Here, A_OL_ and its real-time implementation A_RT_ are trained using a short dataset that necessitates instrumenting subjects with FSRs only once. After training in one reference task, the algorithm detects gait well in post-processing or in real time without operator intervention and for a variety of walking regimes.

We comment that the detection of non-walking in terms of gait phases appears in TD as a general bias towards ST and absence of time spent in the other dynamic phases. In the case of HC, the time in ST may be replaced with time in HO due to the prevalence of toe walking in some patients. The ability of the algorithm to also identify non-walking is an essential feature for wearable orthoses, since the daily routine of a user would necessarily interleave walking and rest periods. Following the instructions to participants, we expect that no time should be spent in SW during non-walking tasks, which is in opposition to the observed results. The nonzero time in SW may be attributed to inaccuracy in the HMM outputs and the inability of participants to complete the tasks as instructed, due to lack of coordination. Nevertheless, we observe less and more time spent in SW and ST, respectively, for non-walking tasks compared to walking tasks, which is consistent with typical gait patterns.

The same gait detection algorithm was implemented for both TD and HC, leaving the training procedure to adapt the HMM parameters to the specific gait pattern of each subject. Defining a unique model for each subject with hemiplegia (for example, toe walking) is expected to lead A_OL_ and A_RT_ to produce higher values of sensitivity and specificity than reported here. The reason for the selected methodology lies in the perspective of implementing this algorithm in an active orthosis designed to progressively induce gait functional tuning. In other words, the algorithm should accommodate a continuously varying training data set in its most general form.

We notice that the smallest values for both sensitivity and specificity occur in non-walking tasks for both TD and HC; this lack of correspondence is most likely due to the training of the algorithm, which used the *L*_1.0_ as input. We expect that this mismatch can be remedied in future incarnations of this work, which will use a variety of training datasets to build a set of models covering everyday tasks. Moreover, the use of FSR data for partitioning gait cycles may be unnecessary for training in later models, as numerical algorithms, such as wavelet analysis, have proven capable of interpreting sensor data measuring posture and gait [20], [34].

### Gait Phases Detected from Gyroscope Data Distinguish between Participants with Pathologies of Varying Severity

Results from A_OL_ are able to distinguish between TD and HC. This fact, combined with the nature of the selected sensors, suggests that such algorithm can be implemented in active orthoses with control schemes aimed at both locomotion and rehabilitation. In some cases, the algorithm is even able to differentiate the more affected leg of the patients with hemiplegia in both walking and non-walking tasks, which lends itself to the necessary implementation of asymmetric leg control in such orthoses.

The development of these active orthoses is particularly important for rehabilitation in children, since age is a crucial factor in the reception of neurological therapy [35], [36]. It is worth noting that this outcome is fundamental in patient-oriented gait training [37]. Moreover, these results highlight inherent differences in the walking and non-walking tasks, wherein populations show more even distributions for the percent of time spent in ST, HO, and SW during walking tasks and higher time in ST during non-walking tasks. This suggests that the HMM methodology may be designed for task identification, similarly to the application in [38].

### Gait Phases Detected from Gyroscope Data may Act as an Assessment Tool

Results from gait detection carried out with A_OL_ may also be used to predict the level of gait ability, evidenced by the negative correlation between GMFM and the percentage of time spent by the patients’ more affected legs in ST and HO. Thus, comparison between a reference distribution of healthy gait phase percentages and data from both of the patient’s legs may also offer an assessment tool to validate measures of motor function and to qualitatively compare the relative function of each leg. We comment that this study has included patients with a wide range of pathology severity; targeting a specific etiology for gait pathology may improve correlation between detected gait phases and motor function in future incarnations of this work.

### The Real-time Implementation is Sufficient to Detect Gait Phases

The close correspondence between the post-processing and real-time implementations of the algorithm indicates that gait detection will be able to be performed in active settings. Since the experiments were 60 seconds long, A_RT_ is able detect the gait phase with a delay that is conservatively computed on the order of a millisecond. Given the characteristic time of human motion, we expect that this time delay does not preclude gait phases detected by A_RT_ as a viable input for real-time gait control [39].

We comment that A_RT_ represents a suboptimal solution compared to the Viterbi algorithm. In fact, the latter is based on a two-step computation of state probabilities for the measured signal, with the first iteration starting from the first sample and proceeding to the last and the second iteration starting from the last sample and proceeding to the first [26]. We define A_RT_ as a so-called left-right model, which uses only the first iteration, since the probability computed by the Viterbi algorithm is highly dominated by this forward step in this type of model. The backward iteration enables refining the state transitions, which we disregard as it also prohibits real-time applications, by introducing significant computational delay. The dominance of results from the forward computation, combined with the 60 ms tolerance window on state transitions, result in the technically suboptimal algorithm implementation A_RT_ providing performance comparable to that of A_OL_.

## Conclusions

In this study, we have developed a novel gait detection algorithm based on a HMM applied to data from a limited sensor set. The algorithm differs from existing models as it is trained on data from a short trial using an operator-independent method, which allows its use on patients with a variety of gait patterns. We have demonstrated that the algorithm can successfully track a FSR reference signal and that a modified implementation enables its use in real time, without significantly affecting results in children both with and without hemiplegia. In addition, this analysis differentiates between children with and without hemiplegia and can be correlated with gross motor functional assessments, which supports its potential as an assessment tool.

Future work on this gait detection algorithm will primarily target replicating the HMM-based algorithm for a range of different activities. In this scheme, a supervising algorithm will treat sensor data with a variety of activity-specific HMMs and identify both the most probable activity from such data and the most probable state within that model. This expansion is expected to enable control schemes for active orthoses which can support patients in a wide variety of everyday tasks, such as walking, running, and climbing stairs. The model input will also be extended to a vectorial form using more diverse sensor placement on the trunk or at multiple leg locations [14], [40] and including salient features of the upper body motion [41].
